# Anti-cancerous effect of albumin coated silver nanoparticles on MDA-MB 231 human breast cancer cell line

**DOI:** 10.1038/s41598-017-05461-3

**Published:** 2017-07-12

**Authors:** Marzieh Azizi, Hedayatoallah Ghourchian, Fatemeh Yazdian, Shahla Bagherifam, Sara Bekhradnia, Bo Nyström

**Affiliations:** 1 Institute of Biochemistry and Biophysics (IBB), University of Tehran, Tehran, Iran; 2 Institute for Cancer Research, Norwegian Radium Hospital, Oslo, Norway; 3 Faculty of New Science and Technology, University of Tehran, Tehran, Iran; 4 Department of Chemistry, University of Oslo, Oslo, Norway

## Abstract

With the aim of making specific targeting of silver nanoparticles as a drug for tumor cells and developing new anticancer agents, a novel nano-composite was developed. Albumin coated silver nanoparticles (ASNPs) were synthesized, and their anti-cancerous effects were evaluated against MDA-MB 231, a human breast cancer cell line. The synthesized ASNPs were characterized by spectroscopic methods. The morphological changes of the cells were observed by inverted, florescent microscopy and also by DNA ladder pattern on gel electrophoresis; the results revealed that the cell death process occurred through the apoptosis mechanism. It was found that ASNPs with a size of 90 nm and negatively charged with a zeta-potential of about −20 mV could be specifically taken up by tumor cells. The LD_50_ of ASNPs against MDA-MB 231 (5 μM), was found to be 30 times higher than that for white normal blood cells (152 μM). The characteristics of the synthesized ASNPs included; intact structure of coated albumin, higher cytotoxicity against cancer cells than over normal cells, and cell death based on apoptosis and reduction of gland tumor sizes in mice. This work indicates that ASNPs could be a good candidate for chemotherapeutic drug.

## Introduction

The discovery and development of new anticancer agents are crucial to prevent side effects and drug resistance problems caused by current available treatments^1^. Clinically treatment of advanced breast cancer is faced with serious challenges, such as dormant micro-metastases, resistance to all systemic therapies, triple-negative breast cancer, genomic chaos, and transformed ER- and HER2-positive breast cancer. Therefore, this type of treatment is not amenable to the targeted therapies, and consequently may cause a progressive increase in symptomatic central nervous system (CNS) relapses that are not controlled by standard monoclonal antibody therapies^2^.

The antimicrobial efficacy of silver nanoparticles (SNPs) has been demonstrated through several studies, although only a few anticancer studies have been conducted in this regard^3–5^. Since the food and drug administration (FDA) approved its usage in human body^4^, SNPs could be used as potential antimicrobial and anticancer agents, especially in emergent situations such as treating burns and healing of wounds^6^.

It is not possible to use every cytotoxic agent for destruction of cancer cells. A chemotherapeutic drug should have the potential to induce apoptosis, as a route of cell death, not necrosis^7, 8^. Apoptosis is a process of programmed cell death, which is used to remove damaged cells^9^. The advantage of apoptosis over necrosis is the lack of a systemic inflammatory response after cell death^10^. The obvious morphological changes that may occur during apoptosis are cell shrinkage, chromatin condensation, extensive plasma membrane bleb, and separation of cell fragments into apoptotic bodies^11^. Apoptosis pathway can be triggered by various pathways and, among which, increasing DNA damage and reactive oxygen species (ROS) generation are considered as the major pathways^9, 12, 13^.

Albumin protein is a dominant drug carrier in serum, which has a variety of binding sites for a large number of drugs^14^. Albumin nanoparticles have recently attracted interest of pharmacologists as anticancer drug carrier systems^15^. Indeed, these nanoparticles make specific targeting of drugs to tumor cells possible; this leads to less toxic effects on non-cancerous cells by enhancing endocytic uptake of drugs via two mechanisms^15^: successful passive targeting of drugs to tumors^16^ and activation of albondin/glycoprotein 60 (Gp60) that mediates albumin transcytosis in endothelial cells^17^. Because of the higher rate of metabolism in cancer cells, the albumin uptake also proceeds by these cells^18^. As such, an albumin-carried drug appears to be absorbed by cancer cells more than by normal cells.

In the present work, with the aim of making specific targeting of SNPs as a drug to tumor cells and development of new anticancer agents, a novel nano-composite was developed named “albumin coated SNPs” (abbreviated as ASNPs). Furthermore, the cytotoxic properties of ASNPs and their anti-cancerous effects were investigated on the most invasive cell line of human breast cancer and white blood cells as normal cell control.

## Results

### Characterization of ASNPs

**Figure 1:**
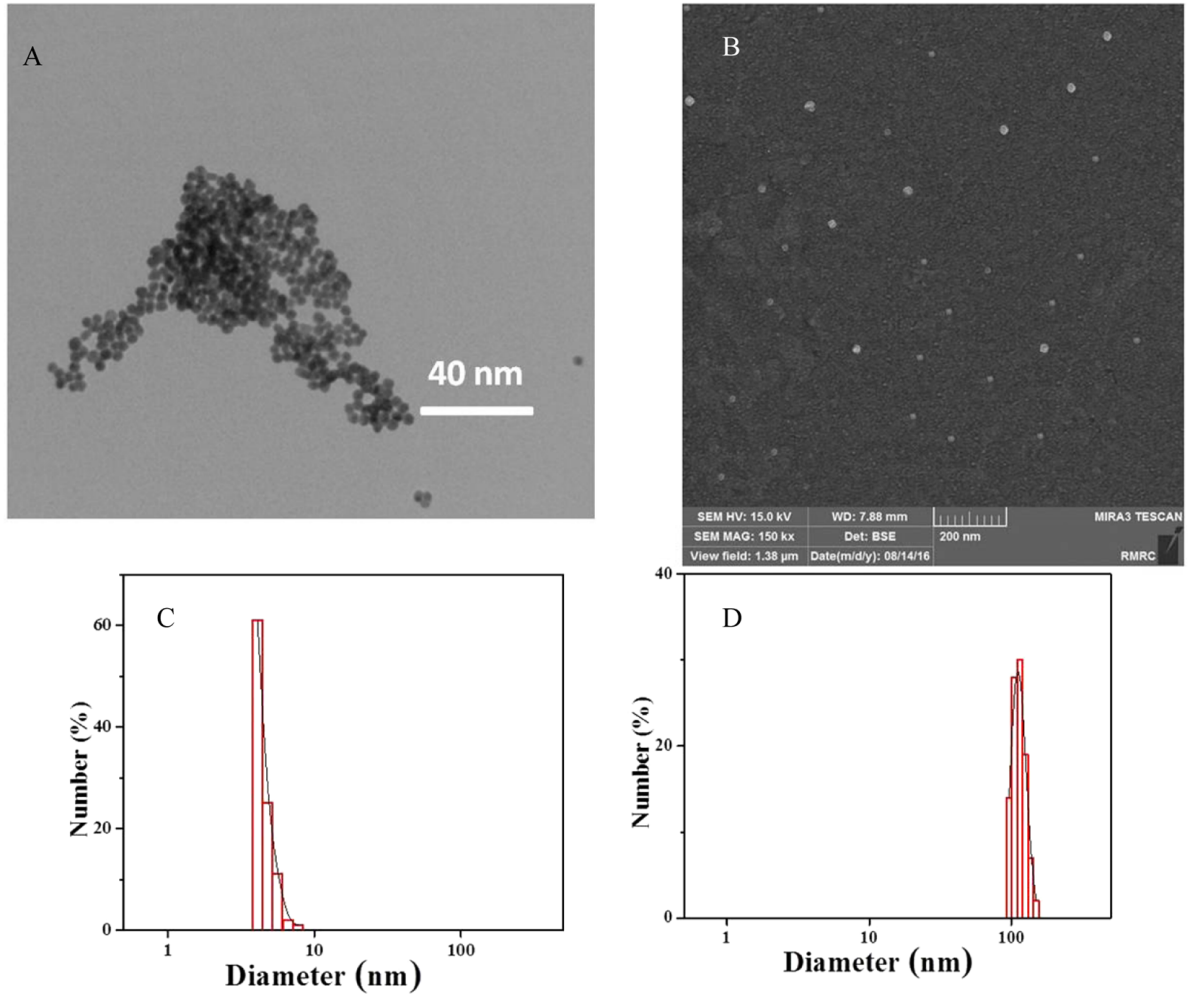
(**A**) TEM image, (**B**) SEM image, (**C**) DLS size distribution of SNPs, (**D**) DLS size distribution of ASNPs.

**Figure 2:**
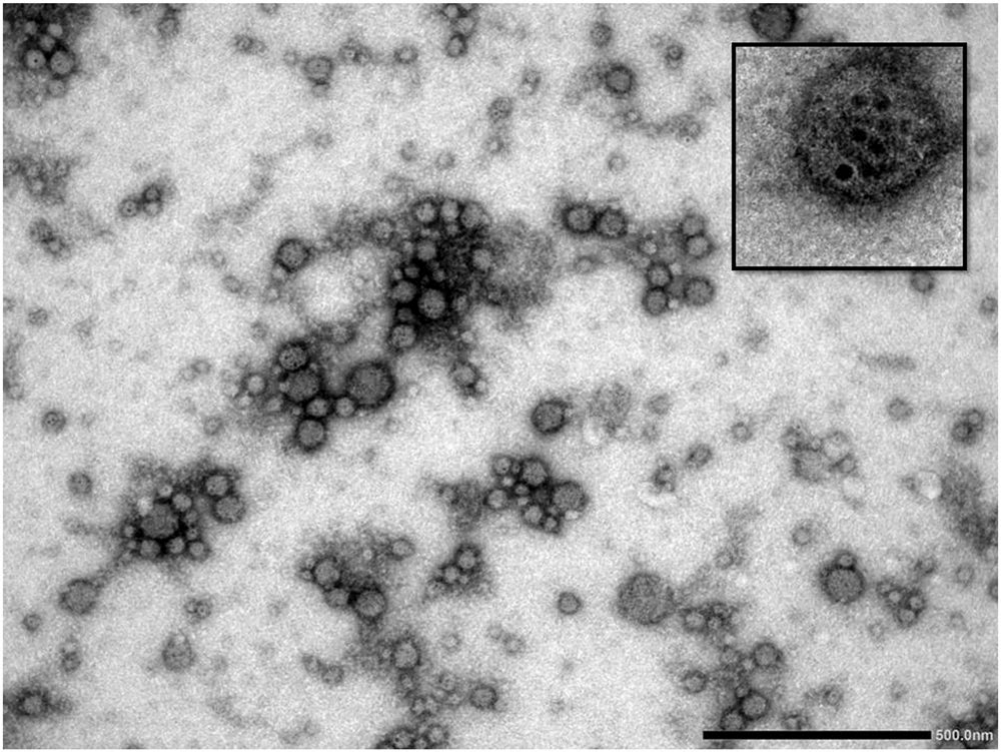
TEM image of negatively stained ASNPs with magnification of 50000 (insert image magnification: 100000).

TEM and SEM images of SNPs (Fig. 1A and B) revealed SNPs were formed spherically and the particle-size histogram obtained by DLS (Fig. 1C) indicated that SNPs vary in size from 4.1 to 7.7 nm with mean diameter of 4.6 nm. TEM images of ASNPs (Fig. 2) revealed spherical shape of ASNPs with rather smooth border and particle size around 100 nm. SNPs inside BSA are seen as black spots because they are metallic particles with a high electron density. As seen, many of the particles contain the black spots but some of them seem to be empty. This indicates that the distribution of SNPs imbedded inside the complexes is not homogenous. There were the most of the clustered conformations that probably formed during the drying procedure in the preparation of samples for TEM also it implied their aggregation behavior. The statistical data regarding the size of the ASNPs based on TEM image (Fig. 2) showed: 1- for p < 0.002, size of nanoparticles is 90 nm with n = 37 numbers, that parameter of n refers to numbers of nanoparticles in this slice, 2- for p < 0.03, size of nanoparticles is 120 nm with n = 10 numbers, and 3- for p < 0.02, size of nanoparticles is 50 nm with n = 22 numbers. The ratio between the silver embedded ASNPs and empty nanoparticles were: 1- for p < 0.002, r was 60%, that parameter of r refers to ration in this slice, 2- for p < 0.03, r was 32%, and 3- for p < 0.02, r was 43%.

**Figure 3:**
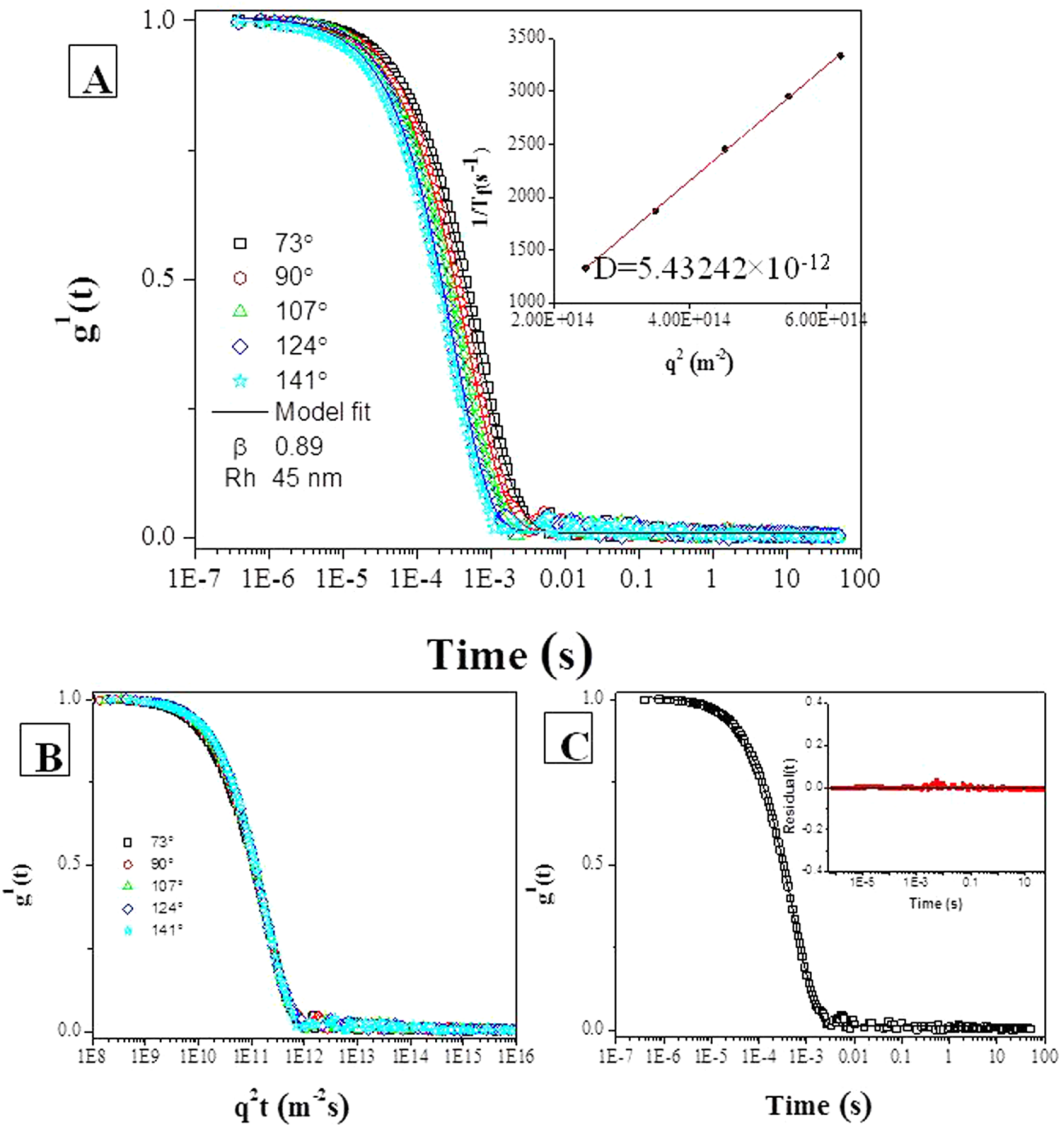
DLS of 1 mM ASNPs in PBS. (**A**) First-order electric field correlation functions versus time, together with curves fitted with the aid of Eq. 1, at the scattering angles indicated: The slope of inset plots (τ^−1^ versus q^2^) is D, which is used to calculate R_h_. (**B**) First-order electric field correlation functions versus q^2^t are plotted at different angles. The condensation of the curves shows the diffusive behavior of the system. (**C**) First-order electric field correlation functions and their corresponding fits at 90°, the inset plot shows the random distribution and small values of the residuals. They indicate that the quality of the fit is good.

Dynamic light scattering analysis was conducted to study the size of ASNPs nanoparticles. Data calculation was based on Eq. 1 (see the experimental part). The decay of the correlation functions was fitted by a single stretched exponential function, and the values of τ_f_ were calculated from Eq. 1a (experimental part). The normalized correlation functions are represented in Fig. 3A. The decay of the correlation function for large particles is shifted to longer time compared to that for small particles. In this case, only one relaxation mode is observed for the ASNPs nanoparticles. The inset plots (1/τ_f_ versus q^2^) show that the relaxation mode is diffusive and we use the Stokes-Einstein relation for calculating the hydrodynamic radii. The slope of this plot yields the apparent mutual diffusion coefficient (D).

In Fig. 2B, g^1^(t) is plotted versus q^2^t for different scattering angels for suspensions of ASNPs (1 mM). The curves collapse onto each other and show the diffusive character of the system. In Fig. 3C, the decays of the correlation functions at an angle of 90° is shown together with its single stretched exponential fit. The inset plot represented the unsystematic distribution and small values of the residuals, indicating that the quality of the fitting is good. The value of β is around 0.89 (>0.8) indicating that the size distribution of ASNPs is fairly narrow. The particle-size histogram obtained by DLS (Fig. 1D) indicated that ASNPs vary in size from 96.5 to 149 nm with mean diameter of 117.4 nm. One albumin molecule has an effective diameter of 7.2 nm^19^ and SNPs (which we synthesized) exhibit diameters around 4.6 nm, therefore every ASNPs with diameter around 117.4 nm contains several SNPs, which are embedded in every cluster of BSA. The common barriers for SNPs (size < 10 nm diameter) delivery to cancer cells and solid tumors is surface opsonization and subsequent entrapment by the mononuclear phagocytic system and reticuloendothelial system, and rapid renal clearance^20^. The pore size of tumor micro-vessels varies from 100 to 1200 nm in diameter^21, 22^. Therefore, ASNPs allows extravasation into tumor tissue but not into normal tissue without opsonization. After synthesizing the ASNPs, the yield percent of the synthesized ASNPs (*y*) was calculated using Eq. 3, to be 97%.

3 $$y=100[{\rm{Weight}}\,{\rm{of}}\,{\rm{ASNPs}}/({\rm{Total}}\,{\rm{weight}}\,{\rm{of}}\,{\rm{BSA}}+{\rm{SNPs}})]$$

### Zeta potential

Zeta potential experiments were conducted to explore the electrostatic stability of the synthesized ASNPs. The magnitude of the zeta potential is representative of the degree of electrostatic repulsion between adjacent and similarly charged particles in dispersion. Small molecules and particles with high zeta potential remain stable, since the solution or dispersion is electrostatically stabilized. For particles with small electrostatic potential, attractive forces, e.g., hydrophobic interactions may exceed the repulsion and the dispersion may flocculate. Therefore, colloids with high zeta potential (negative or positive) are electrostatically stabilized, whereas colloids with low zeta potentials have a tendency to coagulate or flocculate^23^. Furthermore, the release rate and the blood stream circulation and absorption into cellular membranes are quite different. Isoelectric point quantities of positively charged nanoparticles above +10 mV usually lead to rapid clearance from the blood stream. In general, particles with negative charges are less cytotoxic than positively charged ones^24^. The present SNPs possessed a negative charge (−10 mV), which is probably due to citrate anions covering the surface of the particles and thereby prevent them from aggregation. Moreover, encapsulation within BSA nano-carriers still keeps the negative charges, which is probably due to BSA anions (BSA itself has negative charge of −5 mV). Particles with charges from ±10 to ±30 mV are generally found to be stable^23, 25^. In light of this, the synthesized ASNPs in this work with a zeta potential of 19 ± 1 mV (S. Fig.) are expected to be stable.

### UV–Vis spectroscopy

**Figure 4:**
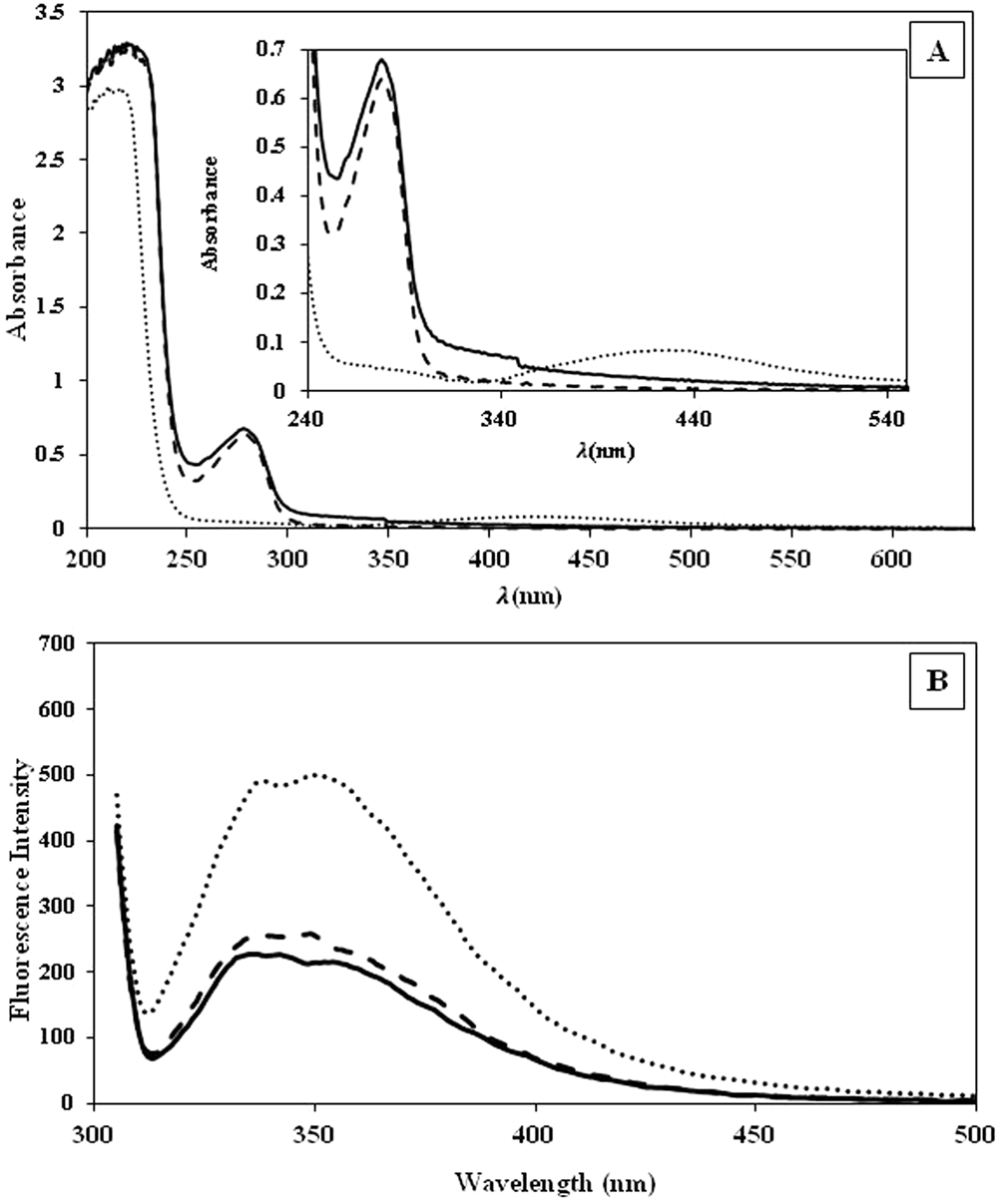
(**A**) UV-Vis spectra of ASNPs (solid line), SNPs (dotted line) and BSA (dashed line). The inset plot shows a magnification of the absorption peak. (**B**) Emission fluorescence spectra of ASNPs (solid line), ANPs (dash-dotted line) and BSA (dashed line).

UV–Vis spectroscopy was used to study the possible conformational changes of the protein. The absorption spectrum of native BSA is characterized by two electronic bands, at 280 nm (due to the phenyl group of tryptophan and tyrosine residues)^26^ and at 210 nm (characteristic peak for polypeptide chains)^27^. The former peak is clearly visible in inset of Fig. 4A. The ASNPs absorption peak position and shape are very similar to that of native BSA in solution (solid line) as shown in Fig. 4A. This suggests that the native structure of BSA was preserved, even after they were transformed to nanoparticle with a high pressure homogenizer. However, a little increment in molar extinction coefficient (hyper chromic) indicates that high pressure homogenization partially condense the protein. Because fluorescent and near-UV spectra both indicated unfolding in the vicinity of tryptophan residues, so the hyper-chromic absorption peak at 280 nm is not due to only tryptophan but may also be ascribed to tyrosine residues, which possibly to some extent are buried in the interior of the protein. The measured UV-visible absorbance spectrum of the suspension of SNPs in PBS (1 mM) is illustrated in Fig. 4A (dotted line) and λ_max_ is centered around 420 nm, which originates from the surface plasmon peak. Since the UV-visible absorption spectra for BSA and ASNPs are matching each other very well, and the plasmon peak has virtually disappeared, these features support the idea that the silver nanoparticles are embedded in the BSA clusters.

### Fluorescence studies

Fluorescence spectroscopy is used to gain information about possible conformational changes in proteins, the extent of hydrophobicity of microenvironment around fluorophores, and the degree of its local mobility^28^. Most of the proteins have some fluorescent amino acid residues, such as tryptophan, tyrosine, and phenylalanine. Excitation at 295 nm in the fluorescence assay gives information about the microenvironment of the tryptophan neighborhood^29^. Each BSA molecule contains two tryptophan residues (Trp-213 and Trp-134)^30^. As seen in Fig. 4B, the emission spectra of ASNPs and ANPs are close to each other, but BSA shows much higher intensity. The decrease of the fluorescence intensity is attributed to quenching of the fluorescence induced by increasing polarity of the micro-environment surrounding the tryptophan residue. Increasing the polarity could be due to: i) the exposure of tryptophan residues to either metallic nanoparticles (SNPs) or ii) unfolding of the local environment of tryptophan residues and the exposure to hydrophilic solvent. This fluorescent reduction is in agreement with increasing the magnitude of molar ellipticity in near-UV spectrum.

### Circular dichroism spectroscopy

**Figure 5:**
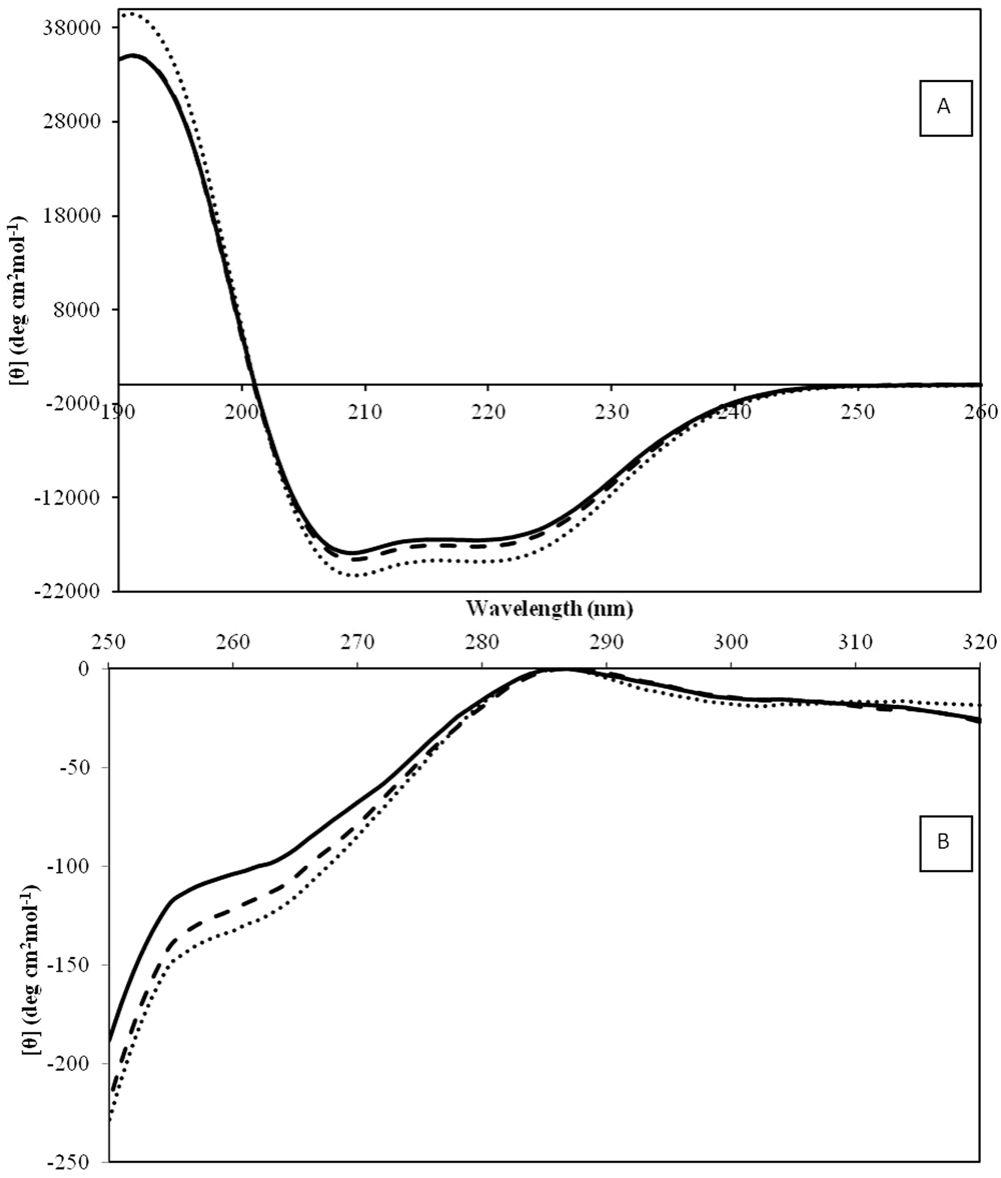
(**A**) Far-UV CD spectra and (**B**) near-UV CD spectra of ASNPs (solid line), ANPs (dashed line-) and BSA (dotted line).

CD spectroscopy was used to evaluate the extent of the conformational changes of BSA in the nanoparticle formation process in comparison with the native protein. The CD spectrum in the far-UV region (190–260 nm) was used to determine the secondary structure changes of BSA. As indicated in Fig. 5A, the CD spectra of BSA in native state, ASNPs, and ANPs exhibited overall spectrum representative of the α-helix structure in protein^31^. It emphasizes that the BSA structure in ASNPs and ANPs are almost similar to its native state. Such a qualitative description was confirmed by spectral deconvolution using CDNN program version 2. As indicated in Table 1, the results are consistent with 1.7% increase in β-strand and 6.1% decrease in α-helix content when the protein is forming complexes with the SNPs. Since α-helix is an indicator for structure stability and native BSA has 66% α-helix, but the α-helix contents in ASNPs and ANPs were reduced 13% and 6%, respectively; we may conclude that the BSA structure during ASNPs formation is intact up to 87%.

**Table 1:** Comparison between the stability percent and secondary structural changes in BSA, ANPs and ASNPs.

	BSA	ANPs	ASNPs
helix	66.4	62.4	58.1
beta sheet	6.3	7.4	8.6
turn	27.3	30.2	33.3
stability	100%	94%	87.50%

Near-UV spectrum from 250 to 320 nm is used to study changes in the tertiary structure. In the near-UV region, the typical CD signals of BSA^32^ were found to be almost conserved for both ASNPs and ANPs (Fig. 5B). The slight peak at 260 nm is the fingerprint of native BSA that is also observed for ASNPs and ANPs, with a negligible decrease in the intensity. These results indicate that in the tertiary structure surrounding tyrosine and tryptophan residues of ASNPs and ANPs, no drastic changes occurred.

### Anticancer evaluation of SNPs and ASNPs

**Figure 6:**
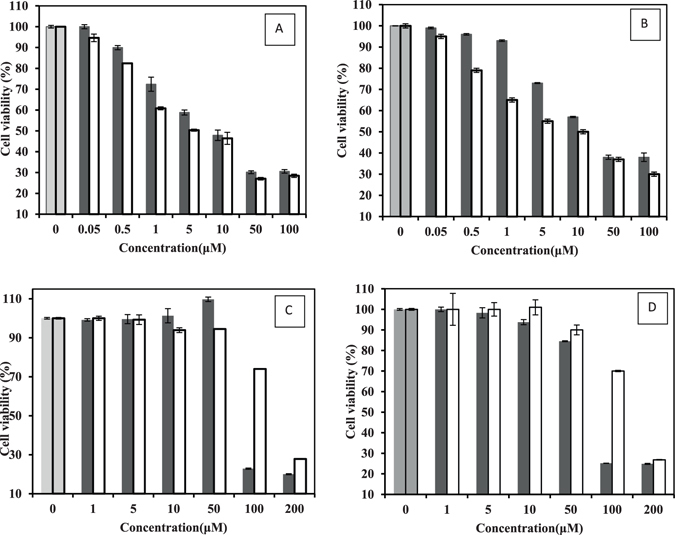
(**A**) Cell viability of MDA-MB 231, (**B**) MCF-7 (**C**) WBC and (**D**) MCF-10A treated with SNPs ■ and ASNPs □. The concentration of nanoparticles is given on the x-axis. The first two columns are the untreated cells as negative control. Each column shows the mean value of three experiments. The error bars were calculated by dividing the standard deviation by the square root of the number of measurements.

An MTT cytotoxicity assay was performed to determine the anti-proliferative effect of SNPs and ASNPs on MCF-7, MDA-MB-231, MCF-10A, and WBCs cell lines. The results showed that both SNPs and ASNPs significantly inhibited the proliferation in breast cancer cell lines of MDA-MB-231 and MCF-7. However, they did not show remarkable effect towards human normal MCF-10A breast cells and normal WBC (Fig. 6). Both SNPs and ASNPs induced dose-dependent cytotoxic effects against different cell types.

It seems from Table 2 that there is a direct correlation between the toxicity of SNPs and ASNPs and proliferation rate of treated cells. This may be rationalized in the following way: First, the highest cytotoxicity was seen for the most aggressive cell line with higher proliferation rate. Second, cytotoxicity of SNPs against breast cancer cells was 3 to 10 times (the magnitude of the difference depends on the type of breast cancer cell line) larger than its cytotoxicity on normal cells. Third, cytotoxicity of ASNPs against breast cancer cells was 14 and 30 times more than its cytotoxicity on normal cells (MCF-10A and WBC). Fourth, the trends of LD_50_ related to both SNPs and ASNPs are as follows: MDA-MB-231 < MCF-7 ≪ MCF-10A < WBC (Table 2). In fact, Table 2 emphasizes that the presence of BSA as carrier of SNPs intensified the difference between the cytotoxicity effect in cancer and normal cells. The reason behind this intensification can be related to enhanced uptake of SNPs via albumin specific receptors (albondin) that mediate albumin transcytosis in endothelial cancer cells^17^. Due to the higher rate of metabolism in cancer cells, the albumin uptake is also increased^18^. As such, the albumin-carried SNPs appear to be absorbed stronger by cancer cells than normal cells. It seems that ASNPs make the specific targeting of SNPs to tumor cells possible, which leads to less toxic effects on non-cancerous cells. On the other hand, the proposed dose of ASNPs (5 µM) is not toxic enough for normal cells to die. Therefore, it seems that the specificity generated by BSA makes the ASNPs a suitable candidate to be used in chemotherapy. It clearly revealed that the uptakes of ASNPs by cancer cells are somehow specific; therefore, it could be an excellent candidate to be used in chemotherapy.

**Table 2:** LD_50_ values of SNPs and ASNPs against MDA-MB 231, MCF-7, MCF-10A and WBC.

Compound	Cell line	Cell type	LD_50_ (μM)
SNPs	MDA-MB 231	an aggressive mesenchymal breast cancer cell line	7
SNPs	MCF-7	a luminal breast cancer cell line	21
SNPs	MCF-10A	normal breast cells	74
SNPs	WBC	normal white blood cell	76
ASNPs	MDA-MB 231	an aggressive mesenchymal breast cancer cell line	5
ASNPs	MCF-7	a luminal breast cancer cell line	10
ASNPs	MCF-10A	normal breast cells	146
ASNPs	WBC	normal white blood cell	152

### Apoptosis induction

**Figure 7:**
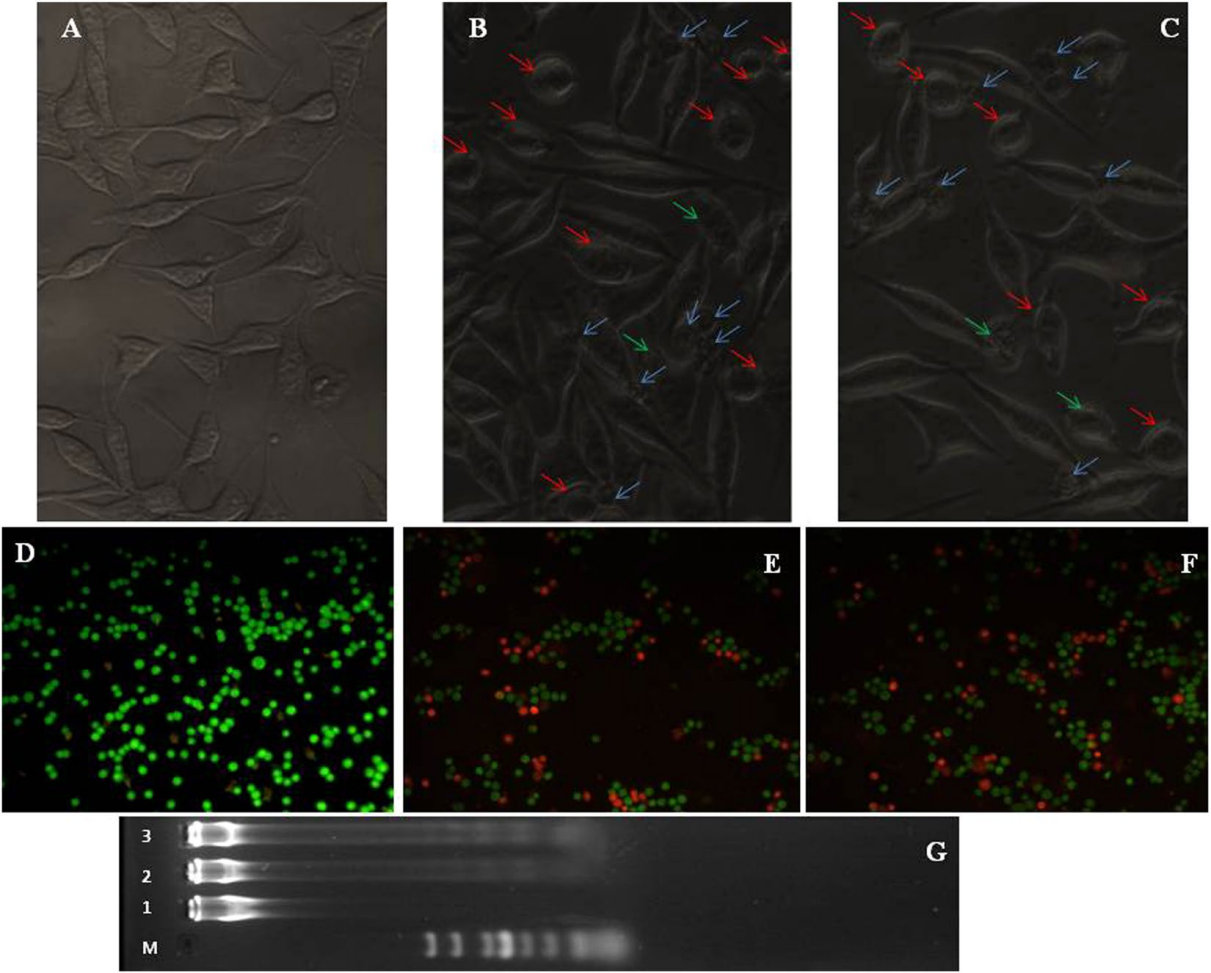
Upper row: Inverted microscopic images of MDA-MB-231 cells treated with LD_50_ concentrations of SNPs and ASNPs for 24 h. (**A**) Untreated cells as control, (**B**) treated cells with SNPs, and (**C**) treated cells with ASNPs. Blue arrows: apoptotic bodies. Red arrows: condense cytoplasm. Green arrows: membrane integrity loss. Intermediate row: Fluorescent microscopy of MDA-MB-231 cells stained with acridine orange/ethidium bromide. (**D**) Untreated cells as control, (**E**) treated cells with SNPs, and (**F**) treated cells with ASNPs. (**G**) DNA ladder on agarose gel electrophoresis. Lane M: 1 kB ladder, lane 1: control, lane 2: SNPs (7 µM) and lane 3: ASNPs (5 µM).

To indicate whether the mechanism of cell death is through apoptosis, MDA-MB 231cells were treated with LD_50_ values of SNPs and ASNPs. Then, the results were evaluated by inverted confocal microscopic images of cells, acridine orange/ethidium bromide staining methods and DNA fragmentation assay. Comparison between the images obtained by inverted microscopy (Fig. 7, images A to C) showed a morphological alteration between treated MDA-MB 231cells by SNPs or ASNPs, and untreated cells. Variations such as loss of membrane integrity, inhibition of cell growth, cytoplasmic condensation, creating curved forms, and apoptosis body were observed in the treated cells. The results indicate that these nanoparticles induce cell death whereas the non-treated cells remained active. The cellular apoptosis was also inspected by a mixture of acridine orange and ethidium bromide staining methods. Acridine orange/ethidium bromide staining was used to visualize nuclear changes and apoptotic body formation that are characteristics of apoptosis. In Fig. 7 (images from D to F), the stained apoptotic cells were compared with the control cells under a fluorescence microscope. The cells whose nuclei are seen as orange spots (images E and F) are apoptotic cells, whereas the viable cells are observed green. The cells treated with ASNPs and SNPs showed an extended apoptosis relative to the control cells. The last step for indicating the apoptosis induction was to compare DNA ladder fragmentation pattern with the products of endonuclease cleavage in apoptosis in the agarose gel^33^. The DNA of MDA-MB-231 cells treated with SNPs and ASNPs at LD_50_ concentrations was extracted and loaded in the agarose gel. The results of DNA “laddering” pattern extracted from MDA-MB-231 cells treated with ASNPs and SNPs are shown in Fig. 7G. Previous studies by Gurunathan and coworkers demonstrated that cancer cell lines treated with silver nanoparticle exhibited the same “laddering” pattern as we observed^34^. It seems that during DNA fragmentation the deposition of silver particles inside the nucleus could affect the DNA and cell division^35^. Perhaps former nanoparticles could induce dose-dependent DNA damage, chromosomal aberrations and errors in chromosome segregation^35^, and formation of sister chromatic exchanges^36^. It has been emphasized that the cell line treated with SNPs induced the production of micronuclei^37^. Therefore, one may conclude that ASNPs can induce DNA fragmentation following the same pathway as mentioned above.

**Figure 8:**
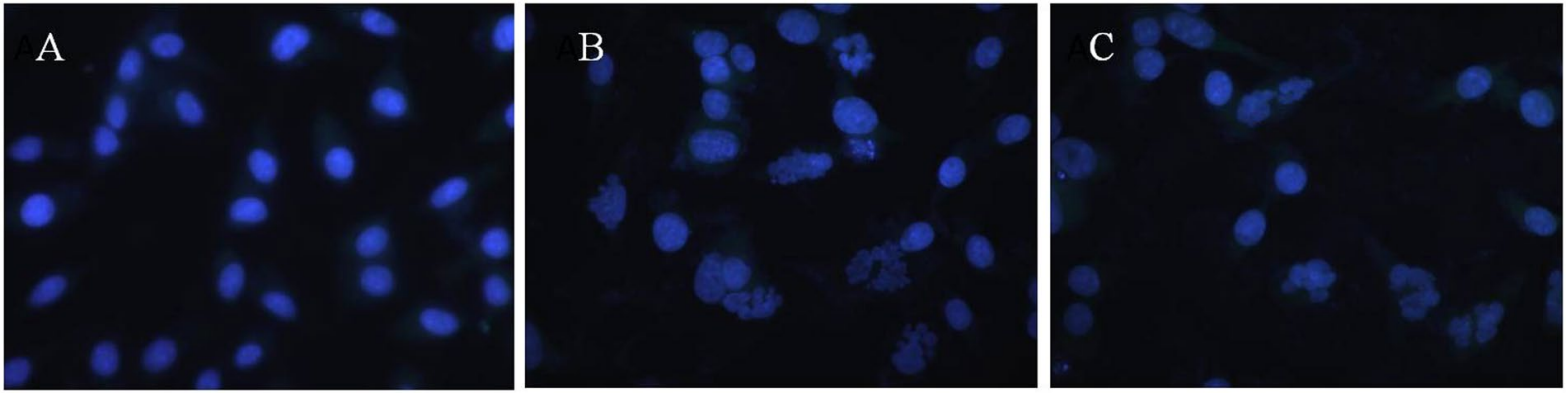
Fluorescent microscopy of MDA-MB-231 cells with DAPI staining. (**A**) Untreated cells as control, (**B**) treated cells with SNPs, and (**C**) treated cells with ASNPs.

To observe nuclear morphology alterations associated with apoptosis more accurately, cells were stained by DAPI after treated with ASNPs and SNPs (Fig. 8). The results indicate that the untreated cells have homogeneous nuclei, whereas ASNPs treated cells like SNPs treated cells shows fragmentized nuclei.

**Figure 9:**
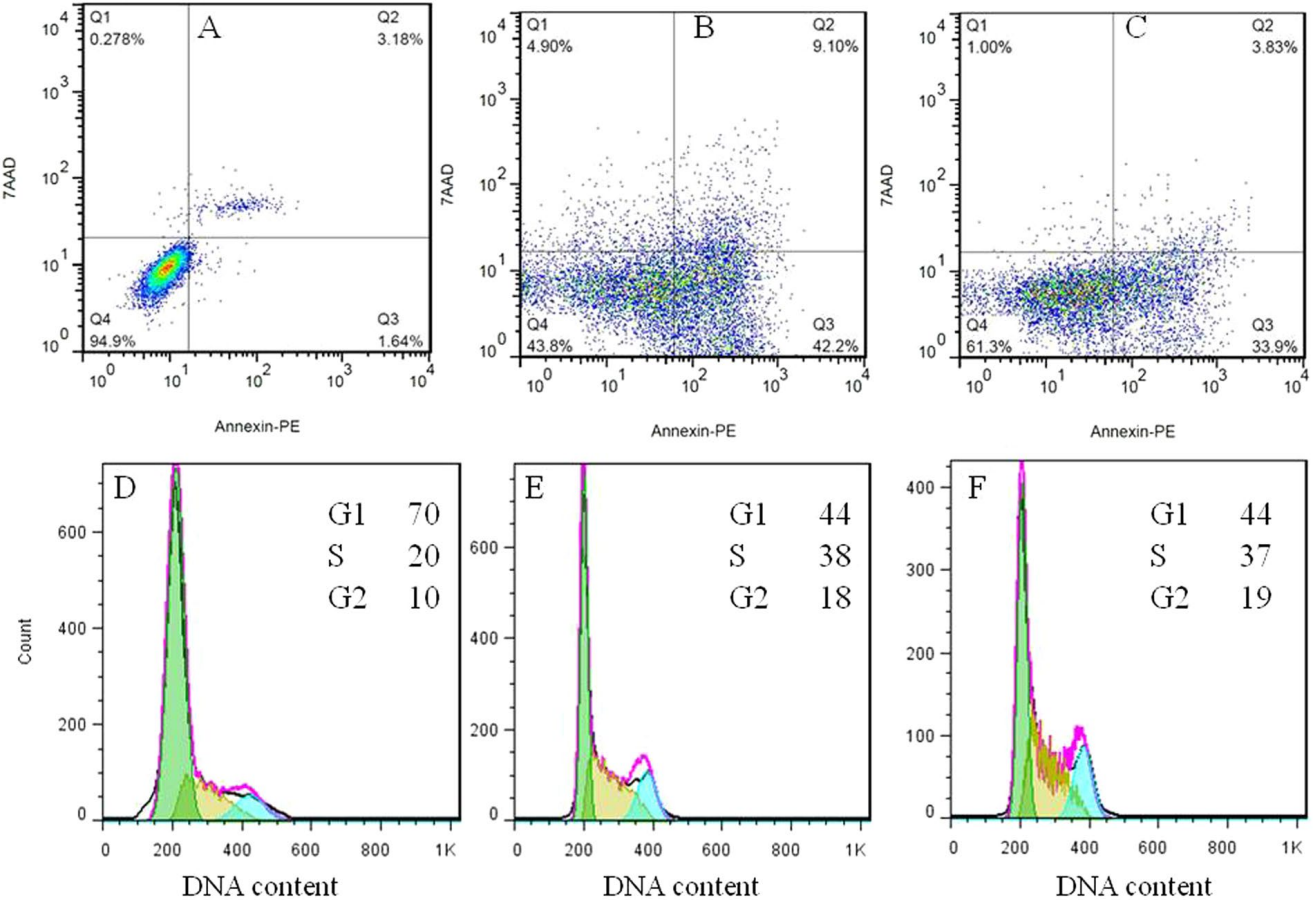
Panels A,B and C represent two-dimensional contour density plots for determination of fractions of early apoptotic, late apoptotic, and necrotic cell death of MDA-MB-231 cells, respectively. Panel A shows the untreated cells as control, while (**B** and **C**) display the treated cells with LD_50_ concentrations of SNPs and ASNPs, respectively, for 24 h. The cell necrosis and apoptosis were measured by using 7-AAD and Annexin-V dyes in flow cytometry based assays. Panels D to F show the effect of ASNPs on the cell cycle. Panel D presents the untreated cells as control, while (**E** and **F**) show the treated cells with SNPs and ASNPs, respectively. The cells were analyzed by flow cytometry, where the cell count versus DNA content is plotted.

**Figure 10:**
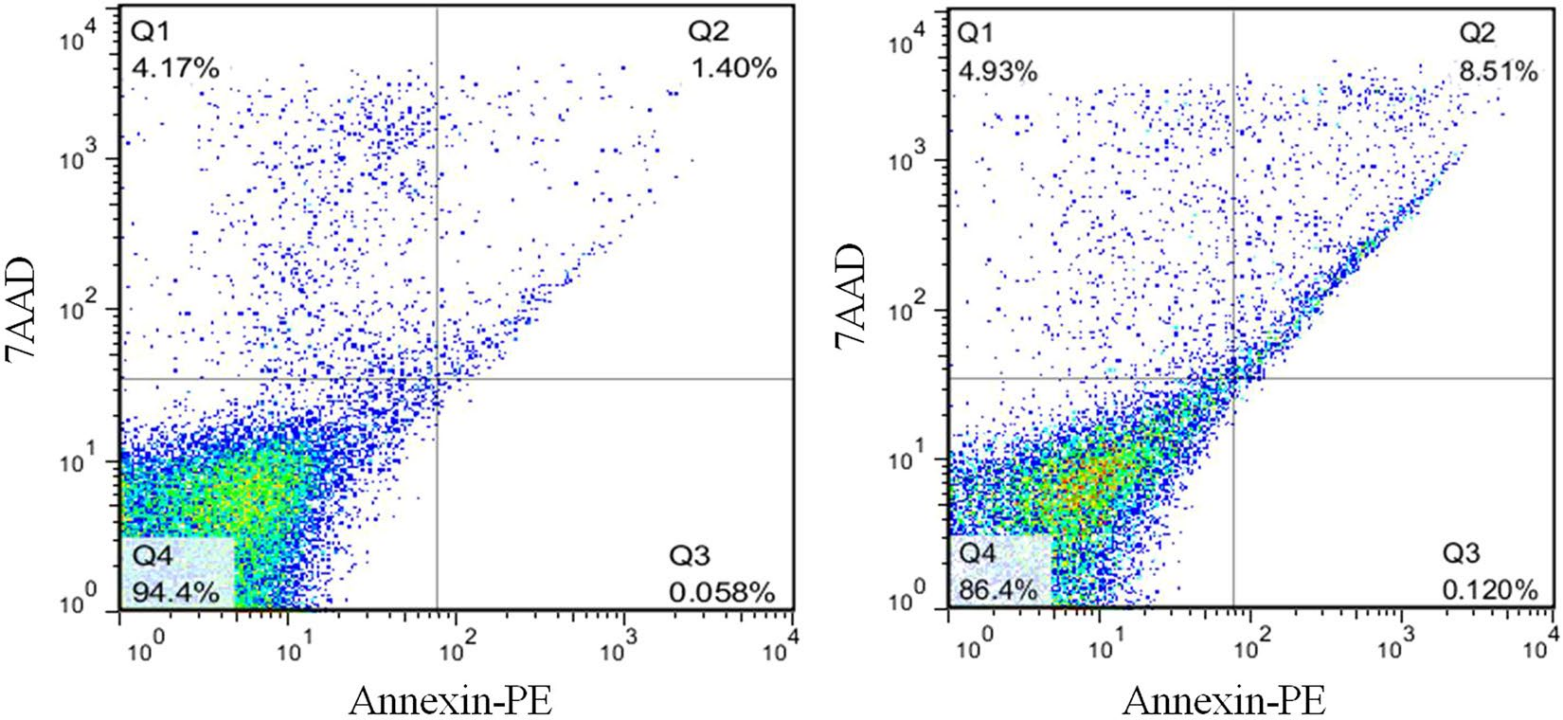
Contour density plots for determination of fractions of early apoptotic, late apoptotic, and necrotic cell death of live WBC cells, respectively. Panel A shows the untreated cells as control, while (**B**)displays the treated cells with LD_50_ concentrations of ASNPs, for 24 h. The cell necrosis and apoptosis were measured by using 7-AAD and Annexin-V dyes in flow cytometry based assays.

To further analyze the feature of cell death induced by ASNPs, we performed apoptosis analysis using flow cytometry. An early marker of apoptosis is the loss of cell membrane phospholipid asymmetry that results in the externalization of phosphatidylserine (PS) bonds on the surface of cells. PE Annexin-V as a fluorescent-labeled Annexin-V dye binds very specifically to externalize PS ligands on the surface of cells leading to the detection of early apoptotic cells. Since externalization of PS occurs in the earlier stages of apoptosis, PE Annexin-V staining can recognize apoptosis at an earlier period than assays based on nuclear changes, such as DNA fragmentation. The loss of membrane integrity, which is preceded by PE Annexin-V staining, accompanies the latest stages of cell death, resulting from either apoptotic or necrotic processes. 7-amino-actinomycin (7-AAD) is a vital dye to allow recognition of early apoptotic cells (7-AAD negative). Viable cells with intact membranes exclude 7-AAD, while the membranes of dead and damaged cells are permeable to 7-AAD. Therefore, cells that are considered viable are PE Annexin-V and 7-AAD negative; cells that are in early apoptosis are PE Annexin V positive and 7-AAD negative; and late apoptotic or already dead cells are both PE Annexin V and 7-AAD positive. Degree of apoptosis in cells exposed to SNPs and ASNPs was quantified by PE Annexin V and 7-AAD staining using flow cytometry. Figure 9(A–C) shows that ASNPs induced apoptosis in MDA-MB 231 cells as well as SNPs, after 24 h treatment by LD_50_ values. However, occurrence of necrosis and lately apoptosis in SNPs treated cells were slightly higher than for ASNPs treated cells. It can be concluded that ASNPs in comparison with SNPs could be a better candidate to use in chemotherapy due to the reduction of the rate of necrosis. On the other hand, to prove that ASNPs are taken up specifically by cancer cells rather than normal cells, WBC cells were treated by 5 μM ASNPs (appropriate concentration in pharmaceutical applications) and the amount of apoptosis and necrosis in normal cells were studied quantitative by the flow cytometry technique. It was verified that 5 μM ASNPs on normal cells increased the apoptosis by 7.22% and necrosis by 0.86% in comparison to untreated cells (Fig. 10). It can be concluded that 5 μM ASNPs has negligible effect on normal cells. Therefore, this is another indication of that ASNPs are specifically taken up by cancer cells rather than normal cells.

Since data obtained by microscopy are qualitative, whereas the results acquired from flow cytometry are quantitative, only the trend of the data obtained from the two methods can be compared. However, to understand the reason why the cell mortality percentages in flow cytometry are higher than those observed by the microscopic method, we have to focus on the principal differences between the two methods. The higher percentage of cell death (apoptosis) in flow cytometry may be due to the asymmetric assay of the cell membrane as an early marker of apoptosis. In other words, PS externalization and loss of cell membrane symmetry is an early and common event during apoptosis of a variety of murine and human cell types, regardless of the initiating stimulus. Externalization of PS proceeds several other events normally associated with the changes in the plasma membrane permeability and even with DNA fragmentation^38, 39^. Since flow cytometry is based on assaying externalization of PS, it can detect cell apoptosis and necrosis before occurrence of other symptoms. Therefore, flow cytometry represents cell mortality of even early apoptotic cells. The microscopic technique can detect the apoptotic cells just after they become permeable to the cell membrane toward fluorescent dye. So, fluorescent microscopy can only detect late apoptotic and necrotic cells, which are less than the whole of apoptotic and necrotic cells.

### Effect of ASNPs on cell cycle

Effects of ASNPs on cell-cycle progression in MDA-MB 231 cells are shown in Fig. 9(D to F). The cell cycle analysis study indicates that the drastic change of the MDA-MB 231cell cycle distributions is the same for ASNPs as for SNPs. 24 h after the ASNPs treatment (5 μM), 44% of the MDA-MB 231 cells were in G1 phase, 37% were in S phase, and 19% were in G2 phase compared to the untreated control cells consisting of 70% of the cells in G1 phase, 20% in S phase, and 10% in G2 phase. A significant decrease in the percentage of cells in the G1 phase and a significant increase in the percentage of cells in the S or G2/M phase was determined for both SNPs and ASNPs treated cells. It has previously been shown that SNPs induces S or G2/M phase arrest at different times after treatment^37, 40^. Data from this project demonstrates that there is not a significant difference between SNPs and ASNPs impact on the cell cycle distributions. This similarity probably suggests that the mechanism of SNPs and ASNPs inside the cells is similar.

Unfortunately, the population of necrotic or apoptotic cells that is distinguishable in apoptosis data (Fig. 9A to C), is not visible in the cell cycle data (Fig. 9D to F). This can probably be related to DNA loss in apoptotic cells, because of cell permeation and fixation in ethanol before staining, which results in the releasing of oligonucleosomes and mononucleosomes. Over 30% of the total DNA exists as low-molecular weight DNA and can be lost in the apoptotic nuclei. Since apoptosis results in a complete fragmentation of nucleus, the low-molecular weight DNA would be able to diffuse outside the cells after ethanol treatment. Furthermore, the DNA stainability of apoptotic cells is reduced because of the activation of endogenous nucleases, followed by the partial loss of DNA. The consequence of these two processes can be under-estimation of apoptotic cell percentage since the apoptotic cells are gated out as debris^41, 42^.

### *In vitro* cellular uptake of ASNPs

Since accumulation of nanoparticles into cancer cells or tumors is of great importance for cancer detection and treatment, a TEM study on thin sections of embedded MDA-MB-231 cells was performed to visualize the presence and confinement within the cancer cells. To confirm whether the ASNPs were internalized into the cells or simply bounded to the surface of the cells, the nanoparticle treated cells were first immobilized, then cross-sectioned and studied by TEM.

**Figure 11:**
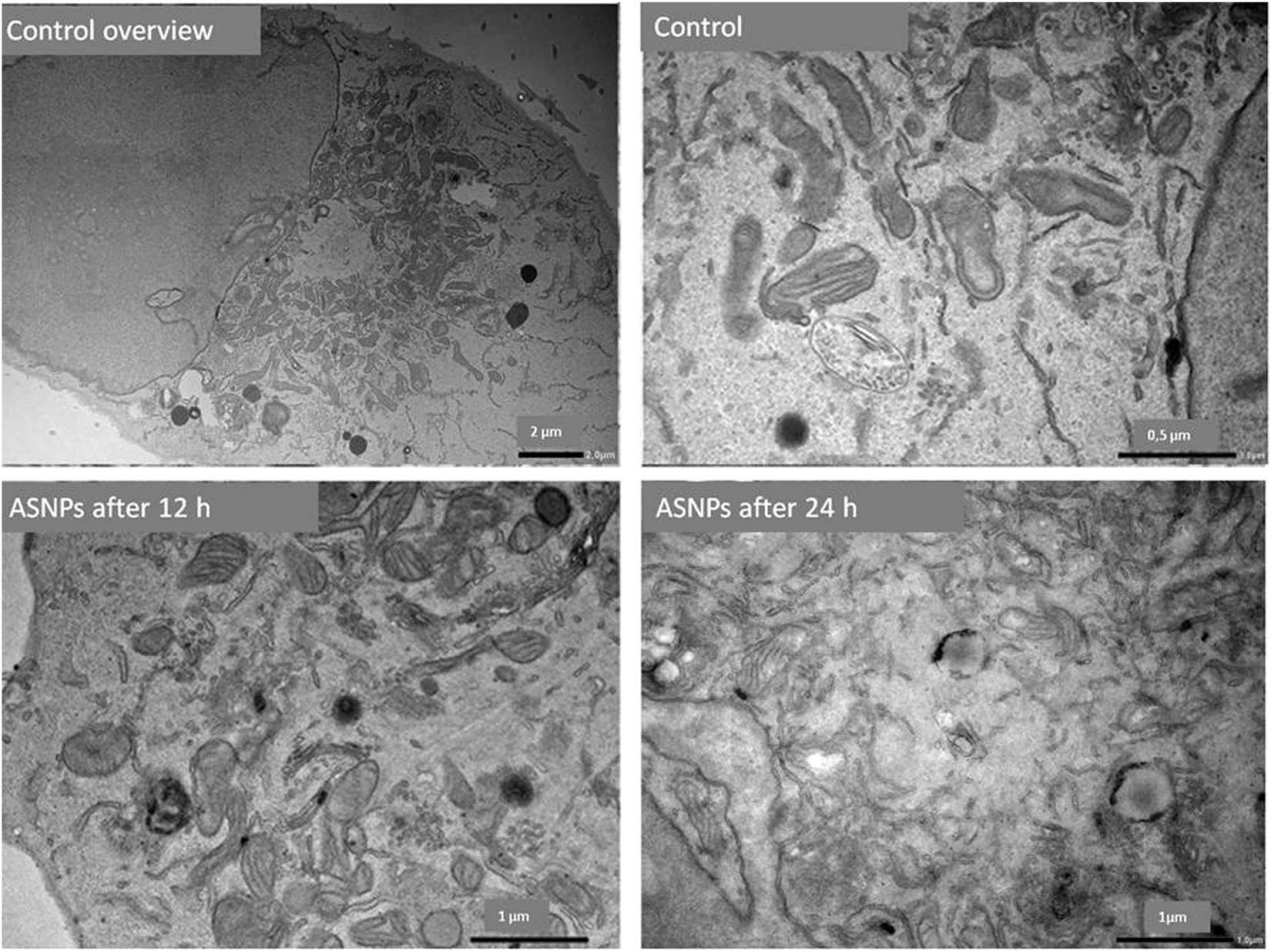
TEM image for internalization of ASNPs across the cell membrane after 12 and 24 hours post treatment.

As seen in Fig. 11, ASNPs crosses the cell membrane and are internalized inside the cells and they are mainly localized close to the mitochondria. This probably occurs via albumin receptor-mediated endocytic pathway. Moreover, as predicted from the cytotoxicity experiments, TEM observations showed that cellular structures were not preserved so that the organelles membrane were destructed in the cells after ASNPs treatment.

It seems that the internalization of ASNPs into the cells may occur via albumin receptor-mediated endocytic two-step process: the first step ASNPs are attached to the cell membrane, via cell surface BSA receptors and the second is the actual internalization of nanoparticles^43^. The condensed black spot around the mitochondria indicates that the ASNPs have lost their BSA coating during the internalization process. By comparing TEM images for treated cells after 12 and 24 hours, it is obvious that the morphology of mitochondria has been changed 24 hours after treatment. This change can be linked to the damage that is caused by interaction of silver nanoparticles with mitochondria^44^. Generally, the crucial tasks of mitochondria involve signaling, cellular differentiation, cell death, maintaining control of the cell cycle, and cell growth. Therefore, according to Kheirollahi *et al*., one may conclude that the SNPs accumulation in the mitochondria plus ROS increment may demonstrate that the mechanism of apoptosis is prompted by ASNPs; this occurs through the intrinsic pathway known as the mitochondrial pathway^45^.

### Drug delivery

**Figure 12:**
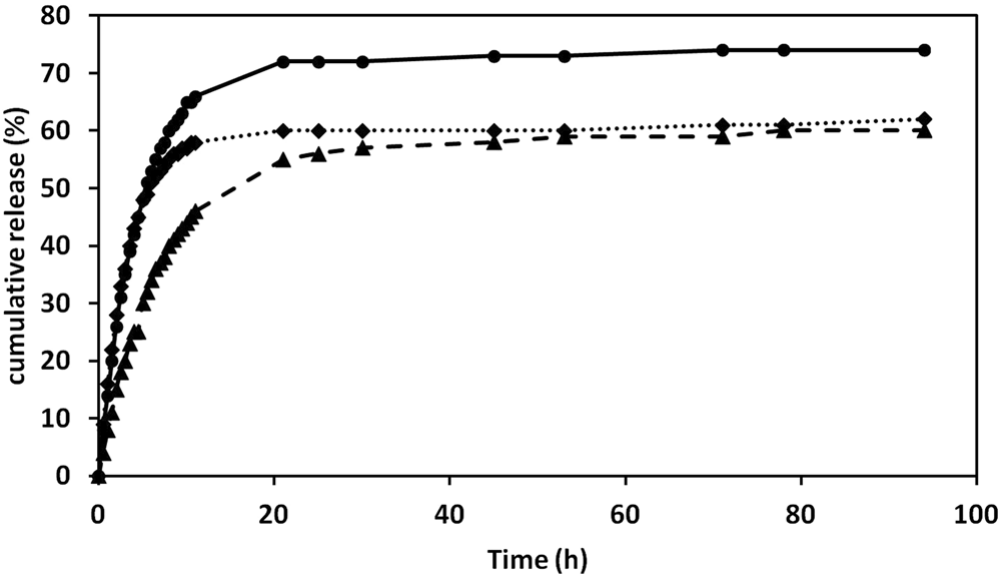
The kinetics of paracetamol release from albumin nanoparticles. The profile of drug release in PBS (pH 7.4) is presented for different concentrations of paracetamol: 5 mg/ml (solid line), 10 mg/ml (dashed line) and 20 mg/ml (dotted line) were used in albumin nanoparticles. Each point is the mean value of three measurements.

A biphasic release pattern of entrapped paracetamol via albumin nanoparticles was observed. Indeed, over a period of 11 h a rapid release of 66%, 46%, and 58% was observed for albumin nanoparticles containing 5, 10 and 20 mg/ml paracetamol, respectively. This stage was followed by a slow and sustained drug release of 74%, 60%, and 62%, respectively, over the next 94 h of the experiment (Fig. 12). The observed initial burst release may be due to the dissociation of surface absorbed drug present in the albumin matrix. In addition, sustained release activity of the drug might be due to the slow release of drug entrapped inside the albumin matrix, as the same pattern has been seen in curcumin-albumin nanoparticles^46^. Furthermore, the release mechanism of drug (prednisolone) from BSA nanoparticles has been shown to be controlled by diffusion and erosion^47^. The constant release of drug in aspirin-BSA nanoparticles has also been demonstrated for prolonged duration (90% at 72 h)^48^. Similarly, 5-fluorouracil-loaded BSA nanoparticles suspension maintained a sustained release of drug for 20 h^49^. In a comparative study performed by Jain and Banerjee, BSA nanoparticles appeared to be promising formulation because of their fairly high zeta potential (>16 mV).

### Effect of ASNPs in cellular ROS

**Figure 13:**
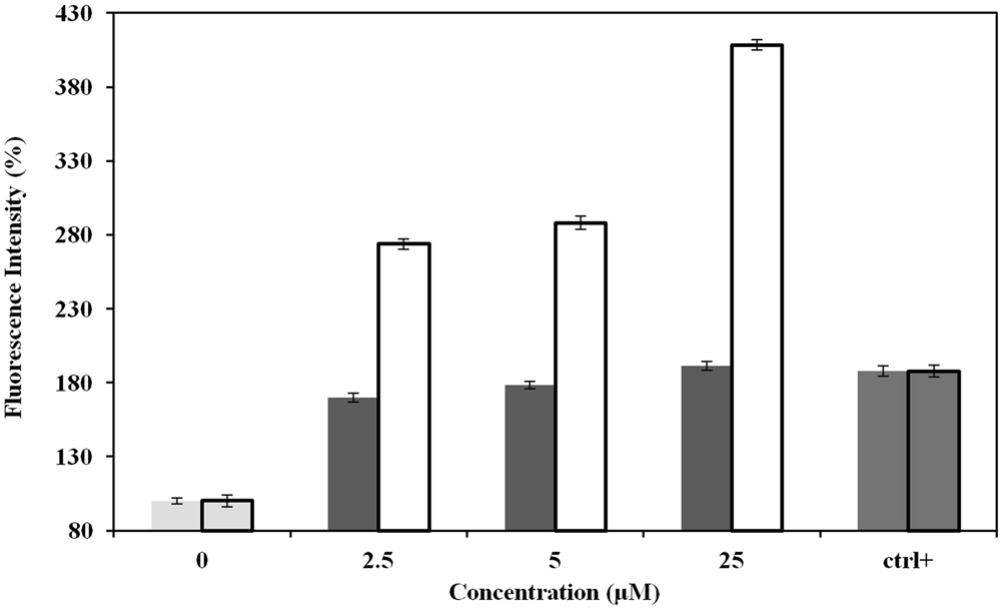
ROS generation in SNPs ■ and ASNPs □ treated MDA-MB-231 cells. Ctrl+ represents positive control cells, which are treated by H_2_O_2_. Relative fluorescence of DCF was measured using a spectrofluorometer with excitation at 485 nm and emission at 530 nm. Each column represents the mean value of three measurements. The error bars were calculated by dividing the standard deviation by the square root of the number of measurements.

To know the effect of ASNPs on oxidative stress, we measured ROS generation using the H_2_DCF-DA assay. The production of ROS leads to disturbed homeostasis in the enzyme system of ROS scavenging antioxidants. As shown in Fig. 13, the ROS levels generated in response to ASNPs treated cells are significantly higher than those for normal cells that are employed as control. These results indicate that cell death is mediated by ROS production, which may alter the cellular redox status. It has been shown that SNPs induce rapidly production of ROS by mitochondria after only 5 and 60 min^50^. This study documented that the smaller SNP particles induced higher levels of mitochondrial ROS. It has been established that increased mitochondrial ROS induces cell death by promoting intrinsic apoptotic pathways^51, 52^. We conclude that ASNPs, as well as SNPs induce apoptosis by this mechanism. Furthermore, it is possible that increased oxidation of DNA is caused by cellular internalization of SNPs. The mechanisms of SNPs internalization are associated with destabilization of cell membranes^53, 54^, but ASNPs use albumin specific receptor for cellular internalization. Therefore, ASNPs induce higher production of ROS in treated cell compared with SNPs treated cells.

### Effect of ASNPs on the body weight of mice and tumor size

**Figure 14:**
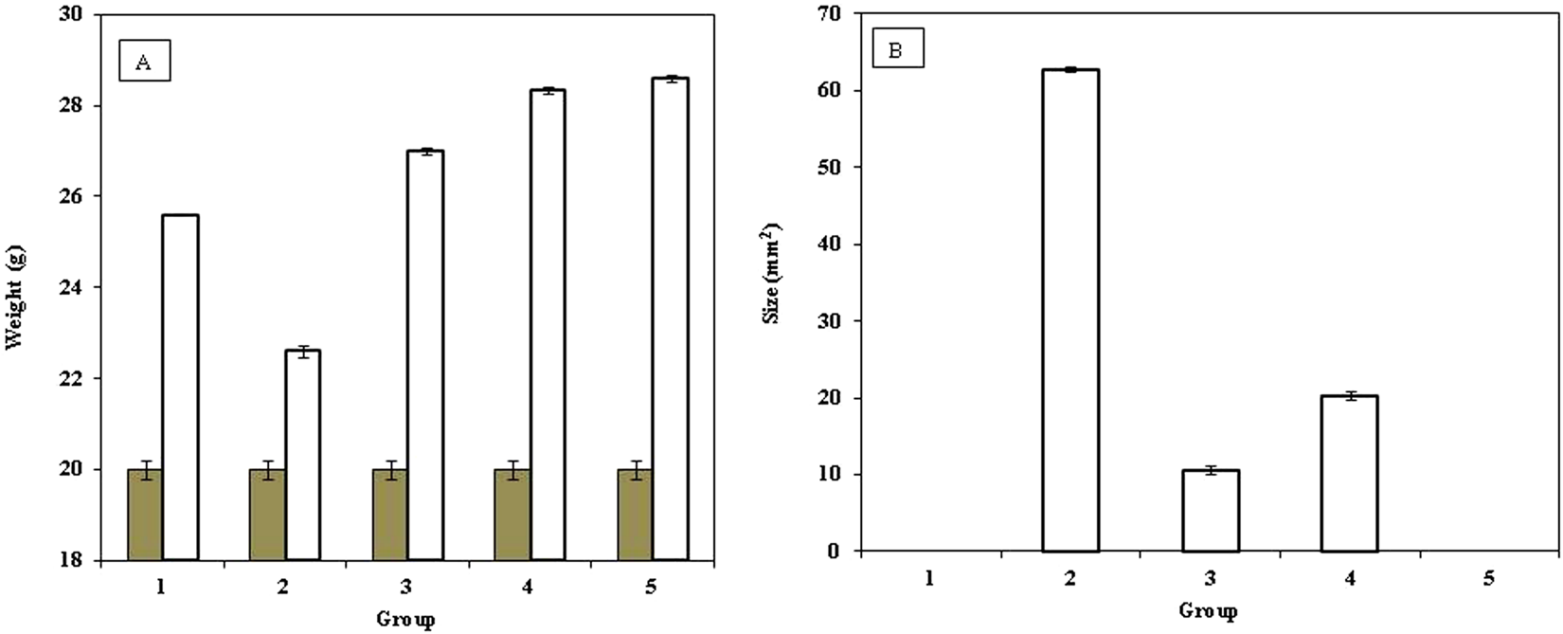
Effect of ASNPs on the: (**A**) mice’s weight as weight of the animal before the start of experiment ■ and at end of treatment □ and (**B**) tumor size in chemically induced murine tumor by 4T1 cells in BALB/c, the results are expressed as the mean ± error bar. Group 1 was untreated mice as control (without 4T1 cells and ASNPs injection), Group 2 was treated only by 4T1 cells, Group 3 was treated by 4T1 cells and after 7 days, when primary tumors reached a mean diameter of 3–4 mm, they were treated by ASNPs, Group 4 was treated by 4T1 cells and ASNPs simultaneously, Group 2 was treated only by ASNPs.

A significant loss of body weight in the tumor-bearing mice is observed (Fig. 14A, Group 2). Both the nanoparticle-treated and non-treated tumor-bearing mice indicated that the treated tumor-bearing mice had gained weight compared with the non-treated tumor group, and the gaining of weight was most pronounced for mice in Group 5, and they were not tumor-bearing mice (Fig. 14A).

A significant difference in the tumor size is depicted in Fig. 14B. As expected, no sign of tumor was observed in groups 1 and 5. The maximum tumor size was found in group 2, which was injected by 4T1 without any ASNPs treatment. Comparison between groups 3 and 4 revealed that ASNPs could prevent tumorigenesis, but this prevention effect is less than apoptosis-induction in tumor. Figure 13 shows that ASNPs treatment removes tumor gland in both groups 3 and 4. The strongest reduction of tumor size occurred in group 3 that is associated with ASNPs-treated tumor-bearing mice.

## Discussion

Anticancer effects of SNPs, especially against the breast cancer cell line of MCF-7 and human laryngeal carcinoma cell line of human epithelial type 2 (HEp-2) are well documented^55^. The proposed mechanism for this effect is interacting of cells and intracellular macromolecules like proteins and DNA can lead to the generation of ROS, which can provoke oxidative stress and apoptosis^56^. Considerable opsonization within a short period of time and rapid renal clearance are the main limitations of using SNPs for treatment for cancer^20^. As such, they led to short plasma half-life time and low drug concentration in the target tissue^57^. On the other hand, albumin based nanoparticles have high potential in targeted delivery of therapeutic agents because of their high drug loading capacity, biodegradability, biocompatibility, and the possibility of covalent or non-covalent binding to different ligands^58^. Therefore, ASNPs were designed and developed for targeted delivery of SNPs to one of the most invasive breast cancer cell lines, namely MDA-MB-231. ASNPs that contain albumin did not show any crystalline structure in the TEM images; therefore, their bioavailability is kept and ASNPs can specifically be taken up by cancer cells through the albumin receptor. The diameter of ASNPs is approximately 90 nm, and the pore size of tumor microvessels varies from 100 to 1200 nm in diameter^21, 22^, allowing extravasation into tumor tissue but not into normal tissue without opsonization. Spectroscopic studies by UV-Vis, fluorescent, far and near CD indicated the least structural changes in the BSA structure. Therefore Gp60 mediates albumin transcytosis^17^ in cancer cells probably would work without problem. ASNPs have a significant anti-proliferative activity against MDA-MB-231 and MCF-7 that showed dose dependent toxicity. However, the LD_50_ value for ASNPs was less than that of SNPs (Fig. 6). This higher cytotoxic activity against cancer cells, which is probably due to higher uptake of SNP to cancer cell, makes less usage of anticancer agent that is advantageous to protect normal cells around cancer cells. Cytomorphological changes observed under inverted phase-contrast microscopy (Fig. 7A to C) and becoming penetrable to acridine orange and ethidium bromide (Fig. 7D to F) and creating ladder pattern in DNA gel electrophoresis (Fig. 6G) after 24 h treatment by ASNPs in MDA-MB-231, all emphasized that the route of cell death in this treatment is apoptosis. Therefore, it possesses the qualification of being chemotherapeutic agent. Furthermore, toxicity on WBCs and MCF- 10 A (Fig. 6B) was also confirmed that ASNPs was 30 times less toxic against normal cells, indicating the specific uptake of nanoparticles to cancer cells and providing further evidence for potential use of ASNPs as chemotherapeutic drug. ASNPs increase ROS production in invasive breast cancer cell line of MDA-MB 231 after 24 h in comparison to SNPs and H_2_O_2_, which may be due to both higher levels of SNPs uptake to the cell and increasing oxidative effect of SNPs with albumin. Anticancer drugs can increase ROS levels in treated cells, and stimulate pro-apoptotic signaling to induce apoptosis^59, 60^. Furthermore, ROS-stressing agents have been proposed as therapeutic strategies to selectively target the destruction of cancer cells^61^. Since ASNPs possess the property of ROS-stressing agents in MDA-MB 231, highlighting the ASNPs as a potential chemotherapeutic drug for invasive breast cancer. At the end *in vivo* data clearly emphasized that ASNPs has a positive effect on growth of BALB/c mice, because group 5 with only drug injection, had higher weight than other. ASNPs had effects on both prevention and treatment of tumor glands, although it had better effect on treatment and removing glands.

## Conclusions

The characteristics of the synthesized ASNPs, such as stability of BSA during the formation of nanoparticle complexes, higher cytotoxicity against cancer cells over normal cells and the cell death based on apoptosis, and the observation that ASNPs can be used as chemotherapeutic drug are important findings in this work. Since the mean diameter of ASNPs is ca. 90 nm and their zeta potential is around −20 mV at physiological conditions, they may have enough stability to interact with cancer cells efficiently. The yield percent of the synthesized ASNPs is high enough, so the process can be scaled up from bench production to clinical and commercial scale. ASNPs were found to reduce the size of tumor gland in mice after induction of tumor and it could prevent induction of gland when tumor induction and ASNPs (as drug) were injected simultaneously. In terms of cost ANSPs are cheaper than current chemotherapeutic drugs. Taken together, ASNPs should be considered as an effective anticancer agent, and this agent is a potential candidate for further pharmacological studies.

## Materials and Method

### Materials

BSA (fraction V, minimum 98%) was purchased from Sigma (Steinheim, Germany). DMEM cell culture and fetal bovine serum (FBS) were purchased from Gibco (United States). 3-(4,5-dimethyl-thiazol-2-yl)-2,5-diphenyl tetrazolium bromide (MTT), 2′,7′-dichlorofluorescein diacetate (DCFH-DA), penicillin, and streptomycin were all obtained from Sigma (United Kingdom), and dimethyl sulfoxide (DMSO) was purchased from Merck (Germany). 7-AAD, Annexin-V dyes, and annexin-V binding buffer were all obtained from BD Biosciences (San Jose, CA). All organic solvents used for TEM were received from VWR, Norway. 4,6-Diamidino-2-phenylindole (DAPI) was purchased from Invitrogen Life Technologies (Eugene, OR, USA). SNPs were synthesized according to the procedure reported by Eskandari *et al*.^62^.

### Synthesis of ASNPs and ANPs

To synthesize ASNPs, a solution (B) was prepared by mixing BSA and SNPs in 1000 μl of PBS/dichloromethane (99/1, v/v). For ANPs, the solution B contained only BSA, and the final concentration of BSA and SNP was 5% and 1 mM, respectively. Another solution A, which was prepared by mixing 20 μl absolute ethanol and 230 μl dichloromethane, was then added to the solution B while the mixture was agitated gently for 5 min. After formation of a crude emulsion it was transferred into a high-pressure homogenizer (Bandelin Sonopuls ultrasonic homogenizer, Germany). The emulsification procedure was done at 20 kHz at room temperature until a milky suspension was obtained. This mixture was transferred into a rotary evaporator, and dichloromethane was rapidly removed at 40 °C at reduced pressure of 30 mm Hg, for 20–30 min.

### Characterization of ASNPs

#### Transmission electron microscopy (TEM)

The morphology of ASNPs was observed by TEM (JEOL JEM 1400 Plus equipped with Ruby camera at 120 kV). A copper grid with formvar and carbon film was positively charged by treating it with glow discharge for 60 s. Then the grid was placed over a 40 μl drop of 5 mgml^−1^ ASNPs suspension for 5 min. The grid was washed with Mili Q water to remove excess of the suspension and then placed over a 20 μl drop of 2% uranyl acetate solution for 2 min and thereafter it was ready for TEM experiments^63, 64^.

#### Dynamic light scattering measurements

Dynamic light scattering (DLS) measurements were performed on an ALV/CGS-8F multi-detector version compact goniometer system, with 8 fiber-optical detection units, from ALV-GmbH., Langen, Germany. The intensity correlation function was evaluated at 8 scattering angles simultaneously in the range 22–141°. The experiments were conducted at 25 °C. In each sample, the experimentally recorded intensity autocorrelation function g^2^(q, t) is directly linked to the theoretically amenable first-order electric field autocorrelation g^1^(q, t) through the Siegert^65^ relationship g^2^(q, t) = 1 + B| g^1^(q, t)|^2^. Where B ≤ 1 is an instrumental parameter and t is time. The wave vector q, is defined as q = 4πn sin(θ/2)/λ; where, λ is the wavelength of incident beam, θ is the scattering angle and n is the refractive index of medium. For our samples, the correlation functions are well described by a single stretched exponential^66^ based on Eq. 1:

1 $${{\rm{g}}}^{1}({\rm{t}})=\exp [-{({\rm{t}}/{{\rm{\tau }}}_{{\rm{fe}}})}^{{\rm{\beta }}}]$$

The correlation functions were analyzed with a nonlinear fitting algorithm to obtain best-fit values of the parameters τ_fe_ and β in Eq. 1. Where τ_fe_ is some effective relaxation time and β = 0.89 (0 < β ≤ 1) is a measure of the width of the distribution of relaxation times.

The mean relaxation time is given by Eq. 2:

2 $${{\rm{\tau }}}_{{\rm{f}}}=({{\rm{\tau }}}_{{\rm{fe}}}/{\rm{\beta }}){\rm{\Gamma }}(1/{\rm{\beta }})$$

where Γ(1/β) is the gamma function of β^−1^.

By assuming spherical shape of the ASNPs, the apparent hydrodynamic radius R_h_ can be calculated through the Stokes–Einstein relationship from the relaxation time (Eq. 3), when the relaxation mode is diffusive.

3 $${{\rm{R}}}_{{\rm{h}}}={{\rm{k}}}_{{\rm{B}}}{\rm{T}}/6{\rm{\pi }}{\rm{\eta }}{\rm{D}}$$

where, k_B_ is the Boltzmann constant, T is the absolute temperature, η is the viscosity of the medium at a given temperature, and the mutual diffusion coefficient D = 1/τ_f_ q^2^.

### Spectroscopy

In all spectroscopic studies (UV–Vis, CD and fluorescence), the concentration of the samples was 40 mM in PBS (0.1 M, pH 7.4). The zeta potential of the ASNPs was determined using a Zetaplus zeta potential analyzer (Brookhaven Instruments) at room temperature. The samples were prepared by diluting the nanoparticle suspension in PBS, pH 7.4. Circular dichroism (CD) spectroscopy was done with the Aviv spectropolarimeter, model 215 (Lakewood, NJ, USA) with a 1.0 cm and 0.1 cm path length rectangular quartz cells controlled by a thermoelectric cell holder (AVIV). Intrinsic fluorescence spectra of the BSA were measured at 25 °C using a Varian Cary Eclipse fluorescence spectrophotometer with excitation and emission slit widths of 5 nm.

### Cell culture

The MDA-MB 231 and MCF-7 human breast cancer cell lines were supplied from the National Cell Bank of Iran (Pasteur Institute of Iran, Tehran, Iran). The MCF- 10 A human breast normal cell line were purchased from Human and animal cell bank at Iranian Biological Resource Center (IBRC). They were cultured in DMEM medium supplemented with 10% FBS, penicillin and streptomycin in a 5% carbon dioxide (CO_2_) cell incubator at 37 °C to reach 70–80% confluence. Human white blood cells (WBCs) were extracted from blood sample of healthy donor by RBC lysis buffer. WBCs were then washed with FBS-free DMEM and adjusted to 1 × 10^4^ cells/well for analysis.

### Cytotoxicity assay

MTT-based assay was accomplished by introductory seeding of 1 × 10^4^ cells (MDA-MB 231, MCF-7, MCF-10A and WBCs) in a 100 μl growth medium in 96-well plate. MDA-MB 231 and MCF-7 cells were treated by increasing concentrations of either SNPs or ASNPs (0, 0.05, 0.5, 1, 5, 10, 50, 100 μM). MCF-10A and WBCs were also treated by either SNPs or ASNPs at the concentrations of 0, 1, 5, 10, 50, 100, 200 μM. Subsequent incubation of treated cells was performed at 37 °C in 5% CO_2_ for 24 h. The half maximal lethal concentration of SNPs or ASNPs (LD_50_) was also calculated as the concentration required for 50% inhibition of cell growth in comparing to the untreated cells^67^.

### Cytomorphological changes of MDA-MB 231

MDA-MB 231 cells were treated with SNPs and ASNPs at LD_50_ concentration and incubated for 24 h at 37 °C in 5% CO_2_ atmosphere. After incubation, the gross morphological changes in the cells were observed under an inverted phase-contrast microscope (Zeiss, Germany).

### Nuclear characteristic of MDA-MB 231

Morphological changes of the cells were studied by acridine orange/ethidium bromide double staining as described in Current Protocols of Immunology^68^. Briefly, cells were cultured in six-well plates (5 × 10^5^ cells/well) and incubated overnight. Cells were then treated with SNPs and ASNPs at LD_50_ concentration, harvested, and washed twice with PBS. Finally, ethidium bromide/acridin orange solution was added to the cell suspension and the nuclear morphology was evaluated by fluorescence microscopy (Zeiss, Germany).

### Determination of nuclear morphology by DAPI staining

The presence of apoptotic bodies and nuclei morphology was examined by DAPI nuclear staining. MDA-MB 231 cells (5 × 10^5^ cells/well) in 12-well plates were exposed to SNPs and ASNPs at LD_50_ concentration for 24 h, then cells were fixed in 4% paraformaldehyde-PBS solution for 15 min and were stained with DAPI (0.3 μmol/l) for 30 min at room temperature. Cells were visualized for nuclear morphology and photography under fluorescence microscopy. Apoptotic cells were recognized and determined based on characteristic observations including the presence of condensed, fragmented, and degraded nuclei.

### DNA fragmentation assay

MDA-MB-231 (10^6^ cells/ml) cells were seeded in 6-well microplates and treated with LD_50_ of SNPs and ASNPs. DNA extraction was done using a salting-out method. Extracted DNA was run in a 2% agarose gel for 20 min by applying 100 V, which was then stained with ethidium bromide. The bands were detected using an ultraviolet transilluminator.

### Cell apoptosis assay

Annexin-V staining was performed to differentiate apoptosis from necrotic cell death induced by ASNPs. Annexin-V protein has a high affinity to phosphotidyl serine, which is translocated from the inner to the outer leaflet of the plasma membrane at early-stage of apoptosis. The conjugation of Annexin-V with the fluorescent probe PE facilitates measurements by flow cytometric analysis. The use of 7AAD staining helps to distinguish between apoptosis and necrosis owing to differences in permeability of 7AAD through the cell membranes of living and damaged cells. Cell number, concentrations, and culture conditions were similar to those used in the cell cycle analysis. Treated cells were harvested and washed twice in PBS. The staining was carried out following the manufacturer’s instruction (eBioscience Annexin V Apoptosis Detection Kit PE, Catalog Number: 88–8102). Cells were incubated in the dark for 15 min at room temperature in 200 µl binding buffer containing Annexin V-PE (10 µl) and 7AAD (5 µl). After incubation, 300 µl binding buffer was added to each sample, and cells were kept on the ice. The 488-nm argon ion laser was used for excitation with BD FACS Calibur (Becton Dikinson, San Jose, CA, USA) flow cytometry system. PE was detected in FL-2, while 7AAD was detected in FL-3. 10000 cells were acquired and data analyses were performed using the Flowjo version 7.6.1.

### Cell cycle assay

Cell cycle analysis was carried out by staining the DNA with propidium iodide (PI), followed by flow cytometric measurement of the fluorescence. Approximately 3.5 × 10^5^ MDA-MB-231 cells per well were seeded in a six-well plate and incubated for 24 h. After the SNPs and ASNPs treatments (LD_50_ concentration of them) for 24 h, the medium was separated and stored. Cells were washed with PBS and trypsinized, then harvested in the stored medium, and centrifuged. The pellet was washed in PBS, fixed in ice-cold ethanol (70%), and stored at 4 °C for at least 12 h. Before flow cytometry analysis, cells were washed in PBS and stained with PI in RNase (40 µg ml^−1^ PI and 100 µg ml^−1^ RNase A) and incubated at 37 °C for 30 min, followed by incubation at 4 °C until analysis. Flow cytometry analysis was performed using FACS Calibur at an excitation wavelength of 488 nm and an emission wavelength of 610 nm. Data collected for 10 000 cells were analyzed using Flowjo software.

### *In vitro* cellular uptake of ASNPs

MDA-MB-231 (10^6^ cells/ml) cells were seeded in 6-well microplates and treated with ASNPs (12 and 24 hours post treatment) the cells were washed with PBS, fixed by 2% glutaraldehyde, post-fixed in 1% osmium tetroxide and dehydrated in alcohol before embedding in Epon. Thin sections (80 nm) of resin embedded MDA-MB-231 cells were cut on a Leica Ultracut UCT. The imaging was performed with a Philips transmission electron microscope CM100 (Philips, Eindhoven, Netherlands).

### Intracellular ROS measurement

The net intracellular levels of ROS generated by synthesized compounds were measured by a non-fluorescence dye, DCFH-DA, which interacts with intracellular ROS to generate fluorescent 2,7-dichlorofluorescein (DCF)^69^. In brief, cells were seeded into 24-well plates at a density of 3.5 × 10^5^ cells per well and were treated with LD_50_ of SNPs and ASNPs for 24 h. Freshly prepared hydrogen peroxide (H_2_O_2_, 0.1 mM in PBS) was used as a positive control in the assay. After treatment, the cells were incubated with 10 μM DCFH-DA for 1 h, washed twice with PBS, and then suspended in 500 μl PBS. The fluorescence intensity was detected, and finally the amount of ROS was subsequently estimated from DCF production.

### Drug release

In the analysis of the mechanism of drug release under physiological conditions, an uncharged substance with intermediate aqueous solubility, paracetamol, was selected as a model drug due to the strong absorption peak at very low concentration. Albumin nanoparticles prepared by the nanoprecipitation technique were loaded with paracetamol as described above. Three different concentrations of paracetamol 5, 10 and 20 mg/ml were used, and the release medium was phosphate-buffered saline (PBS 0.01 M, pH 7.4). Samples were immersed in dialysis tubes (MWCO 1KDa, diameter = 11.5 mm, Spectrum laboratories, Inc., USA) placed into a beaker containing 50 ml release medium. The entire system was kept in a shaker incubator (37 ± 0.5 °C under stirring at 100 rpm). The concentration of paracetamol was then determined in the release medium by an UV-spectrophotometer at 248 nm (extinction coefficient = 165 m^2^mol^−1^) at predetermined time intervals. All measurements were performed in triplicate, and the paracetamol release percentage was obtained according to: Drug release (%) = (D_t_/D_0_) × 100%, where D_t_ and D_0_ indicate the amount of drug released from the nano albumin at certain intervals and the total amount of drug in the nano albumin, respectively. The cumulative amount of drug release over the time period was plotted.

### Animals

Female BALB/c albino mice (six to eight weeks old) were purchased from Tehran Small Animal Research and Teaching Hospital, Faculty of Veterinary Medicine, University of Tehran, Iran. Their body weights were about 20 ± 2.0 g. They were used for experimental purposes with approval from the animal ethics committee of Animal Care and Ethics Committee of the University of Tehran. All animals were housed in polycarbonate cages at 22 ± 2 °C temperature, under medical care, 85% relative humidity and a 12-h light–darkness cycle with standard food and water available ad libitum with pathogen free environment and were allowed to adapt to the laboratory conditions for at least 1 week before surgery. The cages were cleaned at regular interval. All methods and experiments were performed in accordance with the standard ethical guidelines and approved by Animal Care and Ethics Committee of the University of Tehran.

### Experimental design for *in vivo* anticancer study

For the toxicity study, a total of 25 mice were divided into 5 groups, with 5 mice in each group. 4T1, a mammary carcinoma cell line, a highly malignant murine tumor model that resembles advanced breast cancer in human, were used to induce cancer in mice (subcutaneously between mammary gland). The first group was kept as a control without any exposure to 4T1 cells or treatment. Groups 2, 3 and 4 were injected by 1 × 10^6^ cells/100 µl 4T1 cell line. Second group was kept as turmeric control. Groups 3 and 4 were the main treatment groups. Group 3 was treated by 80 µM ASNPs (intraperitoneal injection) when primary tumors reached a mean diameter of 3–4 mm. Group 4 was exposed to a 4T1 cell line and ASNPs together to study prevention role of ASNPs from tumor incidence. Group 5 was treated only by ASNPs.

## Electronic supplementary material

Supplementary figure
