# Supplementary material for: Anti-cancerous effect of albumin coated silver nanoparticles on MDA-MB 231 human breast cancer cell line
Source: Sci Rep. 2017 Jul 12;7:5178. doi: 10.1038/s41598-017-05461-3 (PMC5508052; doi:10.1038/s41598-017-05461-3)
Supplement: Supplementary file 1 — Supplementary figure [file 41598_2017_5461_MOESM1_ESM.doc]

**Anti-cancerous effect of albumin coated silver nanoparticles on MDA-MB 231 human breast cancer cell line**

Marzieh Azizi^1,4^, Hedayatoallah Ghourchian^1*^, Fatemeh Yazdian^3^, Shahla Bagherifam ^2,4^, Sara Bekhradnia ^4^, Bo Nyström^4^

1- Institute of Biochemistry and Biophysics (IBB), University of Tehran, Iran

2-Institute for Cancer Research, Norwegian Radium Hospital, Norway

3- Faculty of New Science and Technology, University of Tehran, Iran

4- Department of Chemistry, University of Oslo, Norway

*Corresponding author: [Institute of Biochemistry and Biophysics (IBB)](http://www.ibb.ut.ac.ir/), [The University of Tehran](http://www.ut.ac.ir/), Tehran, P.O. Box 13145-1384, Islamic Republic of Iran

Tel: +98-21-6640-8920

Fax: +98-21-6640-4680

E. mail address: [ghourchian@ut.ac.ir](mailto:ghourchian@ut.ac.ir)


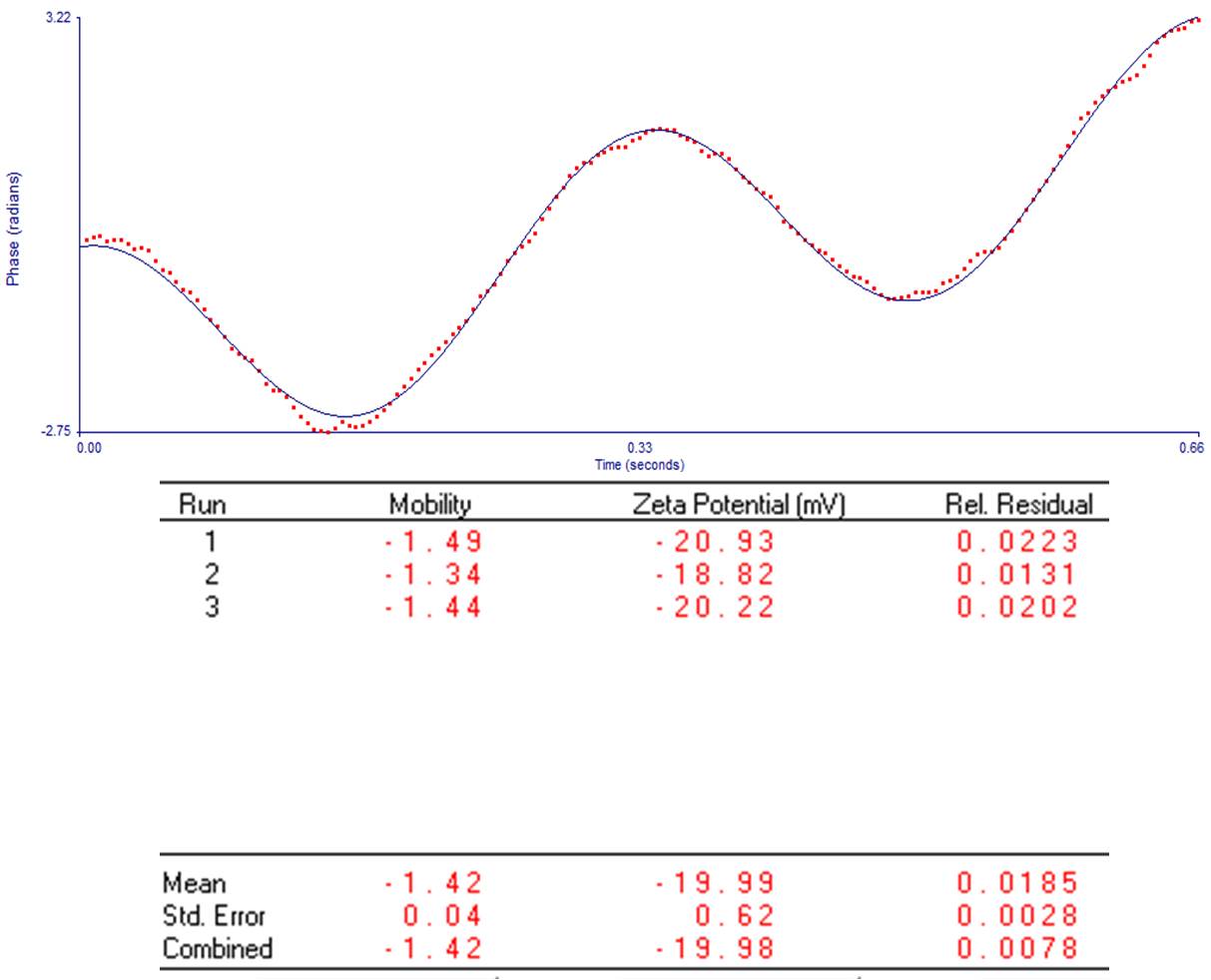


S. Fig. Zeta potential data of ASNPs
